# Supplementary material for: The impact of estimation methods for alcohol-attributable mortality on long-term trends for the general population and by educational level in Finland and Italy (Turin)
Source: PLoS One. 2023 Dec 14;18(12):e0295760. doi: 10.1371/journal.pone.0295760 (PMC10721192; doi:10.1371/journal.pone.0295760)
Supplement: S1 File — (DOCX) [file pone.0295760.s001.docx]

Supporting information 1: data & methods

Description of the data and follow-up design

**Table S1.1 Data sources and available estimations methods for alcohol attributable mortality.**

|  |  | **Finland** | **Italy (Turin)** |
| --- | --- | --- | --- |
| Overall source of data |  | Statistics Finland | Turin Longitudinal Study |
| Follow-up population |  | Finnish population aged 25 and older | Turin population aged 20 and older covered in the population censuses |
| Length of follow-up |  | 5 years | 10 years |
| (Underlying) source of educational data |  | Registers of post-compulsory education certificates (1 Jan 1971, 1976, …. , 2016) | Censuses (24-10-1971, 25-10-1981, 20-10-1991, 21-10-2001, 9-10-2011) |
| (Underlying) source of population at risk |  | Up to 1985 based on census data (1970, 1975, 1980, 1985). After 1985 based on annual population registration. | Population registry of the National Institute of Statistics |
| (Underlying) source of all-cause mortality data |  | Population Information System of the Population Register | Municipality Population Registry of the City of Turin |
| Treatment of immigration |  | Individuals entering Finland during a given follow-up period were included as of the following census. | Individuals entering Turin during a given follow-up period were included as of the following census. |
| Treatment of emigration |  | Individuals who emigrated and did not return during a given follow-up period were excluded from that follow-up period. | For individuals who emigrated and did not return during a given follow-up period, the person-time until their emigration date was counted. |
| Treatment of temporal emigration |  |  |  |
| Source of migration data |  | Up to 1985: census data; From 1986 onwards: annual population registration. | Population registry of the National Institute of Statistics |
| Data based on exact date of birth and death? |  | No. Everyone was assigned a birth date on the 15^th^ of the month | Yes |
| Five-year age groups |  | 30-95+ | 30-95+ |
| Yearly data |  | 1971-2017 | 1972-2019 |
| N of deaths (30+) |  | 2,191,480 | 419,637 |
| N of person years (30+) |  | 142,033,351 | 27,665,912 |
| ICD editions |  |  |  |
| ICD8 |  | 1969-1986 | / |
| ICD9 |  | 1987-1995 | <2014 |
| ICD10 |  | >1996 | >2014 |
| E-codes |  | / | >2014 |
| Estimation methods AAM |  |  |  |
| UCOD |  | 1971-2017 | 1972-2017 |
| MCOD |  | 1987-2017 | / |
| PAF* |  | 1971-2017 | 1972-2017 |

ICD= International Classification of Disease; UCOD = ‘Underlying cause of death, MCOD = ‘Multiple cause of death’, PAF= ‘Population attributable fraction-based; *The PAF estimation method prior to 1990 is based on extrapolated population attributable fractions (see also p.5-6)

Finland

We applied a follow-up of the Finnish population aged 25 and older on 31 December of the (census) years 1970, 1975, 1980, 1985, 1990, 1995, 2000, 2005, 2010, and 2015 during the subsequent five years; or, for the last follow-up period, until the last date for which data are available (31 December 2017). Up to 1985, individual follow-up occurred using data from the official (traditional) census; whereas after 1985, follow-up data stemmed from the annual population registration (i.e. a virtual census).

Those who emigrated during a five-year period and did not return by the end of that same period were excluded. We did so because no exact dates of emigration were available before 1990. Consequently, it was impossible for us to track emigrants more precisely while maintaining comparable definitions of emigration over time. Individuals who temporarily emigrated but came back within the same follow-up period were included for each year in that period (or, if they died, until their date of death), whether living in Finland or abroad. Immigrants entering Finland during a five-year period were not immediately included at their time of entry, but were included as of the subsequent census to ensure that information on their educational level was available.

At the individual level, counting of person days started at the beginning of each year or when a subject turned 30 years old. Counting of person days ended at December 31^st^ each year or on the date of death. After aggregating person-period data by calendar year and age, we ended up with age-period data for individuals aged 30+ from 1971 up to 2017, which we limited to 1972-2017 for this paper.

We obtained remote access to the micro-data through Fiona, and were therefore able to run our analysis scripts on the original data. Because of privacy issues, all individuals in the Statistics Finland data were assigned a birth date on the 15th of the month.

The information on educational level comes from registers of post-compulsory educational certificates, and could be easily translated to the ISCED codes we distinguished for this paper. We determined each individual’s educational level at the start of each five-year follow-up period. In doing so, missing educational information at census indicated that these individuals had no post-compulsory educational certificate. These cases were included in the category “low educated”.

Italy (Turin)

For Italy, we use data from the Turin Longitudinal Study [[1](#_ENREF_1), [2](#_ENREF_2)], which includes demographic, socio-economic, and health information on all individuals who have been resident in Turin (the second-largest city in Northern Italy) once or more often since January 1971. This information was obtained through individual record-linkage procedures. For our research project, we performed 10-year follow-ups of the Turin population aged 20 and older covered in the population censuses in [1971 (24 October), 1981 (25 October), 1991 (20 October), 2001](callto:1971,%201981,%201991,%202001) (21 October), and 2011 (9 October). More specifically, we performed a follow-up from one census until the next census; and, for the 2011 census, we performed a follow-up until the final observation year (31 December 2019).

For individuals who emigrated but returned during a given follow-up period, the person-time for the whole follow-up period was counted (or, if they died, until their date of death). For individuals who emigrated but did not return during a given follow-up period, the person-time until their emigration date was counted. Immigrants were not counted until the following census (i.e. the following follow-up period) to ensure that we had complete information on their educational level.

To enable us to work with full observation years, we defined the study period for Turin as starting on 1 January 1972 and (currently) ending on 31 December 2019. For our analysis, we made use of the exact dates of birth, death, and emigration obtained through the population registers. Similarly, to transform the individual cohort data into aggregate period data, we used the information from the population registers.

The information on educational attainment comes from the different censuses. The Italian qualifications were grouped into the three ISCED categories of low, middle, and high following the ISCED recommendations (UNESCO 2017). Individuals with missing educational attainment were deleted from the follow-up period. The percentage of people with missing educational attainment was less than 1%, except in the last follow-up period, for which the percentage of people with missing educational attainment was around 3%.

Because the Turin Longitudinal Study population is substantially smaller than national populations are, and 1% of causes in the cause-specific mortality data are unspecified (hence reducing the number of deaths that can be used for analysis), we perform two steps prior to calculating alcohol-attributable mortality.

First, we smooth the person years, all-cause, underlying cause of death alcohol-attributable deaths, and PAF-based partly alcohol-attributable deaths using the [Rizzi, Gampe [3]](#_ENREF_3) technique. This technique assumes that ‘binned’ data, such as in 5-year age groups, are indirect observations of finer (i.e. ‘ungrouped’) latent data that can be estimated and used for analyses. In our analysis, we therefore used the R package ‘ungroup’ [[4](#_ENREF_4)] and selected age 100 as the final age for our open-ended age group 95+ from the original data, and estimated the underlying distribution of deaths and person years for single years of age for each year within the study period and by sex and educational attainment . The Rizzi et al-technique thereby uses a penalised composite link model, with the only assumption being that the original distribution is smooth, and it selects the smoothing parameter by minimising the Aikake’s information criterion (AIC) [[3](#_ENREF_3)]. An important property of the technique is that it maintains the observed total death and person-year counts across age. In addition, we proportionally redistributed the year-, sex- and age-specific differences between the original numbers and the smoothed numbers in the total population to the smoothed numbers for the three educational groups, in line with their smoothed shares.

Second, a ratio of all-cause over cause-specific deaths by year, sex, education, and age group is multiplied by the smoothed counts from the previous step to redistribute the potential unspecified deaths that should hypothetically be included in the selected alcohol-attributable causes.

ALCOHOL-ATTRIBUTABLE MORTALITY

Estimation methods

Table S1.2 provides an overview of the causes of death included in each of the methods we used to calculate alcohol-attributable mortality.

Compared to prior research [[5](#_ENREF_5)], we added ICD10 codes K73-K74 (chronic liver disease) to UCOD alcohol-attributable causes of mortality (and, thus, MCOD) to account for ICD revisions and cross-country coding revisions over time. Prior ICD versions included a code that encompassed both cirrhosis and chronic liver disease (i.e. ICD9 code 571), whereas K70 only represents cirrhosis [[6](#_ENREF_6), [7](#_ENREF_7)]. Degeneration of the nervous system due to alcohol (G31.2) was also added to the list of causes considered in prior research.

**Table S1.2. Causes of death (COD) in each alcohol-related mortality estimation method with their ICD-10 codes.**

| **COD** | **ICD-10 code** | **UCOD** | **MCOD** | **PAF*** | **Group** |
| --- | --- | --- | --- | --- | --- |
| Mental and behavioural disorders due to use of alcohol:  -Alcohol-related mental and behavioural disorders  -Degeneration of the nervous system due to alcohol | F10, G31.2 | x | x | x | Other |
| Chronic liver disease and cirrhosis | K70-K76 | K70, K73, K74 | x (-K74.3-K74.5) | x |  |
| Accidental poisoning by and exposure to alcohol | X45 | x | x | x | External |
| Alcoholic cardiomyopathy | I42.6 | x | x | x | CVD |
| Alcoholic polyneuropathy and myopathy | G62.1, G72.1 |  | G62.1 |  | Other |
| Alcoholic gastritis | K29.2 |  | x |  |  |
| Poisoning by and exposure to alcohol, undetermined intent | Y15 |  | x |  | External |
| Transport injuries | V01-99, Y85 |  |  | x |  |
| Self-harm and interpersonal violence | X60-X84, X85-Y09, Y35-Y36, Y87.0, Y87.1, Y89.0, Y89.1 |  | X65 (intentional poisoning by and exposure to alcohol) | x |  |
| Unintentional injuries | W00-X59-X45; Y40-Y84; Y88 |  |  | x |  |
| Epilepsy | G40-G41 |  |  | x | Other |
| Diabetes | E10-E14 |  |  | x |  |
| Pancreatitis | K85, K86 |  | K86.0 | x |  |
| Tuberculosis | A15-A19, B90 |  |  | x |  |
| Lower respiratory infections | J10-J18 (influenza, pneumonia); J20-J22 (other acute lower respiratory infections) |  |  | x |  |
| Ischaemic heart disease | I20-I25 |  |  | x | CVD |
| Stroke | I60-I69 |  |  | x |  |
| Hypertensive heart disease/hypertension | I10-I15 |  |  | x |  |
| Arterial fibrillation and flutter | I48 |  |  | x |  |
| Cancers of the lip, oral cavity, pharynx | C00-C14 |  |  | x | Cancer |
| Oesophageal cancer | C15 |  |  | x |  |
| Colorectal cancer | C18-C21 |  |  | x |  |
| Liver cancer | C22 |  |  | x |  |
| Laryngeal cancer | C32 |  |  | x |  |
| Breast cancer | C50 |  |  | x |  |

UCOD = ‘Underlying cause of death’, MCOD = ‘Multiple cause of death’, PAF= ‘Population attributable fractions-based’, CVD = ‘Cardiovascular disease’, *The partly alcohol-attributable mortality prior to 1990 is based on extrapolated PAFs (see also p.5)

Population attributable fractions (PAFs)

Description and application

In order to estimate deaths partly attributable to alcohol, population attributable fractions (PAFs) for alcohol consumption specific to country-, sex-, five-year age group, year- and cause-of-death combinations were retrieved from the Global Burden of Disease (GBD) study 2017 [[8](#_ENREF_8)]. Yearly sex-, education- and age-specific counts for the causes of death for which an alcohol attributable fraction was retrieved were furthermore extracted from the country-data described above, and subsequently multiplied by their respective PAFs. The resulting numbers were the estimated counts of deaths partly attributable to alcohol, which were combined with UCOD deaths into the “population attributable fraction (PAF)-method for alcohol attributable mortality”, which is thus comparative risk assessment-based.

Alcohol PAFs are positive for most causes of death, implying that alcohol was estimated to cause part of the deaths due to these causes. Nevertheless, PAFs were negative for diabetes mellitus (mainly women), ischaemic heart disease (IHD; women and men aged older than 60), and stroke (women aged 65 and older). However, the idea that alcohol may prevent rather than cause deaths in certain cases is widely debated [[9](#_ENREF_9)]. We therefore do not consider the possibility of negative PAFs in this paper, and instead equate these PAFs to 0.00 when they occur.

Importantly, we averaged the PAFs for lip and oral cavity, nasopharynx, and other pharyngeal cancers because these fractions were relatively similar and clubbing theme facilitated the retrieval of consistent ICD8, -9, and -10 codes from cause-specific mortality data for Finland and Italy.

Potential impact of the PAFs on our findings

Our use of PAFs lead us to consider the following: PAFs are published for the general population, whereby we imply that they are the same for all educational levels. This might affect trends in PAF-based alcohol attributable mortality by educational level to some extent. Indeed, the PAFs for a specific cause of death were applied to individuals in the same sex- and age-specific group in a particular country in a given year, regardless of their educational level. Nevertheless, PAFs would realistically differ by educational level. These fractions are calculated by taking the relative risk of dying for each level of alcohol consumption into account for a specific cause of death, based on how alcohol consumption is distributed in a given country for each combination of age and sex. However, levels of consumption and especially drinking patterns (i.e. frequency, amounts consumed at an occasion, beverage types) generally differ by education, and these aspects are associated with specific health risks [[10-16](#_ENREF_10)]. Furthermore, the risk of surviving any of the conditions included in partly alcohol-attributable mortality may be higher among the lower-educated due to aspects such as differential health care access and comorbidity [[17](#_ENREF_17), [18](#_ENREF_18)]. However, we used the published PAFs because, to date, no socioeconomic status-specific PAFs are included in the GBD Studies, nor Finnish or Italian studies on alcohol-attributable mortality.

REFERENCES

[1] Costa G, Demaria M. Un sistema longitudinale di sorveglianza della mortalità secondo le caratteristiche socio-economiche, come rilevate ai censimenti di popolazione: Descrizione e documentazione del sistema. Epidemiologia e Prevenzione. 1988;36:37-47.

[2] Cardano M, Costa G, Demaria M. Social mobility and health in the Turin longitudinal study. Social Science & Medicine. 2004;58(8):1563-74.

[3] Rizzi S, Gampe J, Eilers PH. Efficient estimation of smooth distributions from coarsely grouped data. American Journal of Epidemiology. 2015;182:138-47.

[4] Pascariu MD, Dańko MJ, Schöley J, Rizzi S. Ungroup: An R package for efficient estimation of smooth distributions from coarsely binned data. Journal of Open Source Software. 2018;3(29).

[5] Mackenbach JP, Kulhanova I, Bopp M, Borrell C, Deboosere P, Kovacs K, et al. Inequalities in Alcohol-Related Mortality in 17 European Countries: A Retrospective Analysis of Mortality Registers. PLoS Medicine. 2015;12(12):e1001909.

[6] Jasilionis D, Meslé F, Shkolnikov VM, Vallin J. Recent life expectancy divergence in Baltic countries. European Journal of Population. 2011;27(4):403-31.

[7] Trias-Llimos S, Kunst AE, Jasilionis D, Janssen F. The contribution of alcohol to the East-West life expectancy gap in Europe from 1990 onward. International Journal of Epidemiology. 2018;47(3):731-9.

[8] IHME. Global Burden of Disease Collaborative Network. Global Burden of Disease Study 2017 (GBD 2017). Results. Seattle, United States Institute for Health Metrics and Evaluation (IHME); 2018 [Available from: <http://ghdx.healthdata.org/gbd-results-tool>.

[9] van de Luitgaarden IAT, van Oort S, Bouman EJ, Schoonmade LJ, Schrieks IC, Grobbee DE, et al. Alcohol consumption in relation to cardiovascular diseases and mortality: a systematic review of Mendelian randomization studies. European Journal of Epidemiology. 2021;37(7):655-69.

[10] Bloomfield K, Grittner U, Kramer S, Gmel G. Social inequalities in alcohol consumption and alcohol-related problems in the study countries of the EU concerted action 'Gender, Culture and Alcohol Problems: a Multi-national Study'. Alcohol and Alcoholism. 2006;41 (Supp 1):i26-36.

[11] Hall W. Socioeconomic status and susceptibility to alcohol-related harm. The Lancet Public health. 2017;2(6):e250-e1.

[12] Hupkens CL, Knibbe RA, Drop MJ. Alcohol consumption in the European Community: uniformity and diversity in drinking patterns. Addiction. 1993;88(10):1391-404.

[13] Jones L, Bates G, McCoy E, Bellis MA. Relationship between alcohol-attributable disease and socioeconomic status, and the role of alcohol consumption in this relationship: a systematic review and meta-analysis. BMC Public Health. 2015;15(1):400.

[14] Kuntsche E, Rehm J, Gmel G. Characteristics of binge drinkers in Europe. Social Science & Medicine. 2004;59(1):113-27.

[15] van Oers JA, Bongers IM, van de Goor LA, Garretsen HF. Alcohol consumption, alcohol-related problems, problem drinking, and socioeconomic status. Alcohol and Alcoholism. 1999;34(1):78-88.

[16] Wood S, Bellis M. Socio-economic inequalities in alcohol consumption and harm: evidence for effective interventions and policy across EU countries. Brussels: European Union. 2017.

[17] Mäkelä P. Alcohol-related mortality as a function of socio-economic status. Addiction. 1999;94(6).

[18] Mäkelä P, Keskimäki I, Koskinen. What underlies the high alcohol related mortality of the disadvantaged: high morbidity or poor survival? Journal of epidemiology and community health. 2003;57(12).
