# Supplementary material for: The impact of estimation methods for alcohol-attributable mortality on long-term trends for the general population and by educational level in Finland and Italy (Turin)
Source: PLoS One. 2023 Dec 14;18(12):e0295760. doi: 10.1371/journal.pone.0295760 (PMC10721192; doi:10.1371/journal.pone.0295760)
Supplement: S2 File — (DOCX) [file pone.0295760.s002.docx]

Supporting information 2: enhanced underlying cause of death method

Summary

We developed a new method to estimate alcohol-attributable mortality (AAM) by educational level based on the advantages and disadvantages of existing methods in the context of studying AAM by education or educational inequalities in AAM, and on the comparison of trends, levels and age-distributions in AAM, both for the general population and by educational level, of the available methods.

Three important observations form the basis of our novel estimation method. First, trends in age-standardised AAM are largely identical for the two approaches that rely on cause-of-death-information only (UCOD, MCOD), but rather different for the PAF-method(Figs S2.1 and 2.2). Second, age patterns in AAM are similar for the three methods until age 65, but become largely different for the wholly-partly method at older ages (Figs S2.3a and b). Third, levels in AAM seem proportionally lower for the underlying cause of death (UCOD) approach compared to the Multiple cause of death (MCOD) approach and the Population attributable fraction (PAF) approach between ages 30-64 (Figs S2.3a and b). In line with these observations, our novel estimation method uses UCOD AAM as its basis, but corrects for known underestimations therein. More specifically, this novel method assumes a similar trend and age pattern compared to UCOD, but adjusts the age-specific levels of AAM upwards so that it matches the age-specific levels for the PAF method between ages 30-64.

We implemented our novel method separately by country, by (1) calculating yearly sex-specific ratios between the age-standardised PAF and UCOD AAM rates for the ages 30 up to 64; (2) averaging these ratios over the different years; and (3) applying these average ratios to the UCOD death counts, by sex, education and age (30+).

Applying this new method yields age-specific and age-standardised mortality rates for Italy that start off lower (Figs S2.4 and S2.5b), and are similar to the MCOD rates in Finland (Figs S2.4 and S2.5a). In both countries, relative inequalities (using the relative inequality index) in AAM are similar for enhanced UCOD and UCOD (and MCOD in Finland), while they are lower using the PAF-method (Fig S2.6 and S2.7). Absolute inequalities (using the slope index of inequality) were overall higher using UCOD-Enh. and PAF (and MCOD in Finland), than using UCOD as the estimation method for AAM.

Rationale for a new method

Existing estimation methods for AAM have benefits and limitations for the study of trends by educational level. We therefore based a new method on the existing benefits and drawbacks, as well as on a visual comparison of existing methods.

The basics and limitations of existing estimation methods

For European research on AAM, the following estimation methods have previously been used in either in cross-country or single country studies:

- - **Underlying cause of death** (‘UCOD’), corresponding to a list of alcohol-specific causes of death mentioned as the underlying cause. Includes wholly AAM only.
  - **Multiple cause of death** (‘MCOD’), corresponding to a specific list of alcohol-specific causes of death mentioned as the underlying or one of the three first contributory causes of death. Includes both wholly and partly AAM.
  - **Population** **attributable fractions (PAF)-** method^[[1]](#footnote-1)^, that considers conditions that are either wholly are partly attributable to death. The total death count due to alcohol is thereby calculated as the sum of Deaths x PAF for each relevant cause, whereby PAFs for wholly alcohol attributable conditions are equal to 1.00 (identical to the UCOD-method) and PAFs for partly attributable conditions are lower than 1.00 according to the Global Burden of Disease (GBD) Study [2]. Importantly, PAFs were only published for the years 1990-2017 at the time of writing. Supporting information 1, Table S1.2 contains details on the causes of death included in each of these methods.

However, no method for AAM is perfect and each is known to suffer from important limitations when it comes to studying trends in AAM in multiple countries (by educational level). First, despite its wide availability and stability, and its reliance on individual death certificates that are therefore education-specific, the **UCOD** severely underestimates AAM because it does not consider any conditions that are partly alcohol-attributable [3]. Second, although the PAF-method includes both wholly and partly AAM, the AFs used to estimate it are not education-specific, and are limited to the period 1990-2017. They furthermore suffer from the same limitations as the data they are based on, being that they rely on non-country- or education-specific meta-data on relative risks of dying by each level of alcohol consumption by age and sex in a given population (compared to abstainers). These consumption data generally come from surveys and are often of lower quality at higher ages and likely represent higher-educated groups better due to sample selectivity [4]. Finally, although also including both wholly and partly AAM from individual (thus education-specific) death certificates, the extent to which the **MCOD**-method captures older-age diseases that may in part be attributable to cancer is questionable (e.g. most cancers) [5]. Contributory cause-of-death information is furthermore not always available.

These attributes of existing methods lead us to consider the MCOD-approach the least flawed for the specific purpose of studying AAM over time by educational attainment, but the required contributory cause-of-death data is not available in some countries, such as Italy to date.

We therefore aim to develop a method that can serve as an alternative when no extensive data on causes of death or education-specific PAFs is available.

Main principles of the new estimation method

We developed our new method based on known limitations and the careful study of how three existing estimation methods for AAM in terms of their levels, age patterns, and trends.

Figs S2.1 and S2.2 display trends in age-standardised mortality according to these methods for the general population. First, rends in age-standardised AAM are largely identical for the COD-approaches (i.e. UCOD and MCOD), but differs for the PAF-method in Finland. In Italy (Turin), trends are similar (i.e. exponential decline) regardless of the method used (i.e. UCOD, PAF).

Second, Fig S2.3a shows that the age pattern in AAM for the three methods in Finland is largely similar up to age 65, but PAF strongly diverges from the COD-based methods at older ages. This similarity followed by divergence is also visible between PAF and UCOD in Turin (Italy) (FigS2.3b).

Third, levels in AAM are visibly lower for UCOD in both countries compared to PAF –and in Finland, also the MCOD – methods, both at the age-specific and age-standardised level (Figs S2.1-3). The level differences between methods is thereby seemingly proportional (on a log scale) between age-specific UCOD rates on the one hand, and (MCOD in Finland and) PAF on the other hand in both countries between ages 30 and 64.

In line with these observations, our novel estimation method uses UCOD AAM as its basis, but corrects for known underestimations therein. The novel method thereby assumes a similar trend and age pattern as observed for UCOD, while adjusting its age-specific levels upwards to resembles those of the PAF method between ages 30 and 64. This age delineation is applied due to known inaccuracies in attributable fractions for ages 65 and older, which PAF relies on. Indeed, alcohol attributable fractions are calculated using information about alcohol use at all ages, which is limited at old ages; age-specific relative risks (RR) of dying at different levels of alcohol consumption are lacking at those ages, and more general evidence about the influence of alcohol on health at older ages is lacking [4, 6]. Janssen, El Gewily [7] also avoided the use of PAF-based age-specific rates over age 65 and instead relied on UCOD age patterns (not levels) at older ages.

Our novel estimation method was implemented separately by country and sex, with the specific steps taken to obtain our adjusted rates described below.

Application

Given the observations for different estimation methods in the data, our method is designed to increase the levels in UCOD with a ratio-based approach that captures the relationship between wholly and partly alcohol-attributable mortality between ages 30-64, hence correcting for known underestimations in alcohol-attributable mortality using UCOD.

The data required to calculate the number of AAM deaths according to our new method consists of death counts according to the UCOD and the PAF methods by country, sex, educational level, and age.

First, we calculate directly age-standardised mortality rates according to the UCOD and PAF methods in the general population between ages 30 and 64, using the European Standard Population 2013 [8]. We subsequently calculate the ratio of these PAF and UCOD AAM rates by country, sex and year between the years 1990 and 2017 [equation 1]. We do so to ensure we only use information about published (and not extrapolated) PAFs in the GBD 2017 study, which the PAF method relies on. Second, we calculate the average of these yearly ratios by country and sex over the 1990-2017 period. These yearly and average ratios can be found in Table S2.1. Third, we apply the obtained country- and sex-specific average ratio to the country-, sex-, education-, and age-specific UCOD death counts for ages 30 and over, thus obtaining the new ‘Enhanced UCOD’ alcohol attributable death counts [equation 2]. The counts for the general population by country, age, and sex are obtained by summing the education-specific deaths.

[1] ${Ratio}_{c,s}=\sum_{y} (\frac{{ASDR{3064}_{PAF}}_{country,sex}}{{ASDR{3064}_{UCOD}}_{country,sex}})/N_{y}$

[2] ${D\_UCOD\_Enh}_{c,s,e,a}= {D\_UCOD}_{c,s,e,a} \times{Ratio}_{c,s}$

**Table S2.1** **Yearly and average ratios of the UCOD and PAF age-standardised mortality rates for ages 30-64.**

|  | **Finland** | | **Italy (Turin)** | |
| --- | --- | --- | --- | --- |
| Year | Males | Females | Males | Females |
| 1990 | 2.38 | 2.30 | 3.09 | 2.72 |
| 1991 | 2.46 | 2.41 | 3.77 | 4.79 |
| 1992 | 2.55 | 2.30 | 3.74 | 2.98 |
| 1993 | 2.53 | 2.31 | 3.85 | 2.53 |
| 1994 | 2.47 | 2.35 | 3.29 | 3.54 |
| 1995 | 2.29 | 2.39 | 4.16 | 3.99 |
| 1996 | 2.05 | 2.10 | 3.65 | 3.87 |
| 1997 | 2.19 | 2.03 | 3.60 | 3.42 |
| 1998 | 1.94 | 1.87 | 3.81 | 3.04 |
| 1999 | 2.04 | 1.87 | 3.11 | 2.79 |
| 2000 | 1.96 | 1.93 | 3.64 | 2.95 |
| 2001 | 1.97 | 1.81 | 3.93 | 4.16 |
| 2002 | 1.96 | 1.91 | 4.21 | 2.59 |
| 2003 | 2.02 | 1.77 | 3.07 | 3.35 |
| 2004 | 1.83 | 1.69 | 2.92 | 2.67 |
| 2005 | 1.81 | 1.66 | 3.18 | 2.82 |
| 2006 | 1.78 | 1.63 | 2.97 | 2.80 |
| 2007 | 1.69 | 1.58 | 2.99 | 3.27 |
| 2008 | 1.69 | 1.53 | 2.96 | 3.90 |
| 2009 | 1.69 | 1.64 | 2.68 | 2.90 |
| 2010 | 1.76 | 1.65 | 2.55 | 3.11 |
| 2011 | 1.78 | 1.54 | 3.13 | 3.59 |
| 2012 | 1.66 | 1.58 | 2.85 | 4.38 |
| 2013 | 1.69 | 1.60 | 380 | 2.58 |
| 2014 | 1.67 | 1.66 | 3.42 | 4.93 |
| 2015 | 1.75 | 1.58 | 3.93 | 3.93 |
| 2016 | 1.82 | 1.64 | 3.99 | 2.93 |
| 2017 | 1.88 | 1.70 | 3.85 | 2.66 |
| Average | 1.98 | 1.86 | 3.43 | 3.33 |

Observations and considerations

The newly developed Enhanced UCOD (UCOD-Enh.) method provides an alternative to estimate alcohol-attributable mortality by educational attainment when limited data is available (e.g. only underlying COD data and published PAFs), given its estimation of both wholly and partly AAM while relying on as much education-specific data as possible.

UCOD-Enh. rates are highly similar to the available MCOD rates among Finnish females (1987-2017), and are slightly lower among Finnish males (Fig S2.4). Indeed, we based the increase in UCOD for our new method on the similarities between the age-specific PAF and MCOD levels for ages 30-64 and because both of these two methods consider partly alcohol attributable mortality in some way. These age-specific rates are more similar among females and somewhat lower for PAF than MCOD among males (Fig S2.3a). At the age-specific level (Fig S2.5a), our new method also shows these lower UCOD-Enh. versus MCOD rates among Finnish males, while rates for Finnish females are virtually the same.

In Italy, the UCOD-Enh. rates lie below the PAF ones (Fig S2.4). Importantly, however, given the small number of deaths identified for Italian females – particularly at the end of the study period -, age-standardised rates tend to fluctuate more year to year. Due to the ratio-based approach of the UCOD-Enh. method, trends in these new rates will rely heavily on the stability in the UCOD baseline. Smoothing of the UCOD death counts may be advisable prior to calculating the UCOD-Enh. deaths and/or rates in smaller study populations.

With regards to trends in inequalities in AAM, the Enh.-UCOD method duplicates the RII observed in UCOD by using this as a baseline and applying a ratio to it, but absolute inequalities are higher according to UCOD-Enh. than UCOD (Figs S2.6 and 2.7). In Finland, we find increases between the late 1980s and early 2000s, followed by a decline for males but less so among females (Figure S2.6). In Italy(Turin), absolute inequality reductions were substantially higher according to UCOD-Enh. compared to the UCOD method. Also compared to the PAF approach, improvements in mortality inequalities would be slightly more emphasised using UCOD-Enh..

Figures

**Fig S2.1.** **Trends in age-standardised alcohol attributable mortality rates according to the available estimation methods by country and sex for the general population aged 30 and older, Finland and Italy (Turin), 1972-2017.** UCOD = ‘Underlying cause of death’, MCOD = ‘Multiple cause of death’, PAF = ‘Population-attributable fractions-based’; Rates are expressed per 100,000 person years. The Y-axis scale differs by sex to improve visibility of the results Data sources: Statistics Finland, Turin Longitudinal Study.


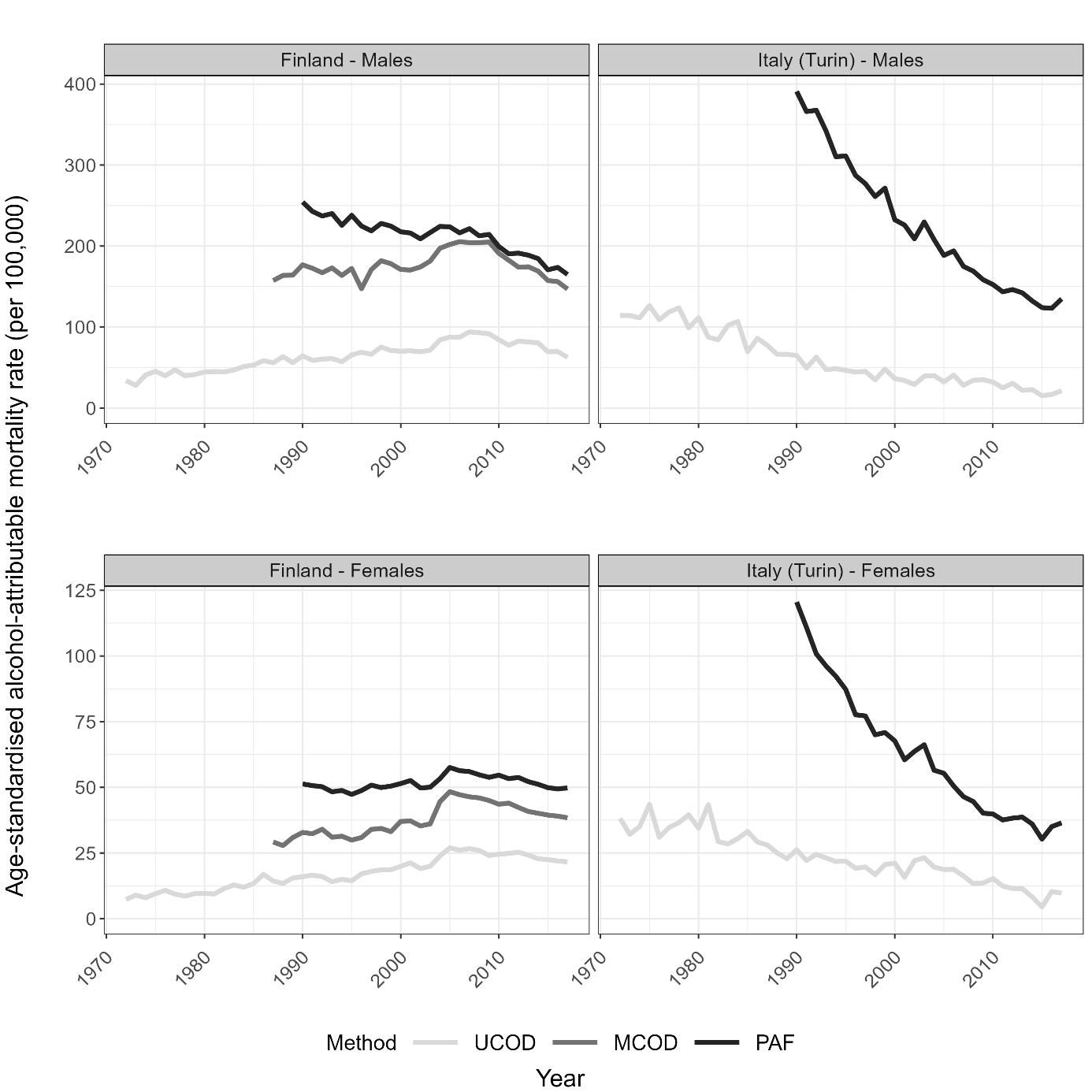


**Fig S2.2.** **Trends in age-standardised alcohol attributable mortality rates according to the available estimation methods by country, sex and educational level for those aged 30 and older, Finland and Italy (Turin), 1972-2017.** UCOD = ‘Underlying cause of death’, MCOD = ‘Multiple cause of death’, PAF = ‘Population-attributable fractions-based’; Rates are expressed per 100,000 person years. The Y-axis scale differs by sex and country to improve visibility of the results. Data sources: Statistics Finland, Turin Longitudinal Study.


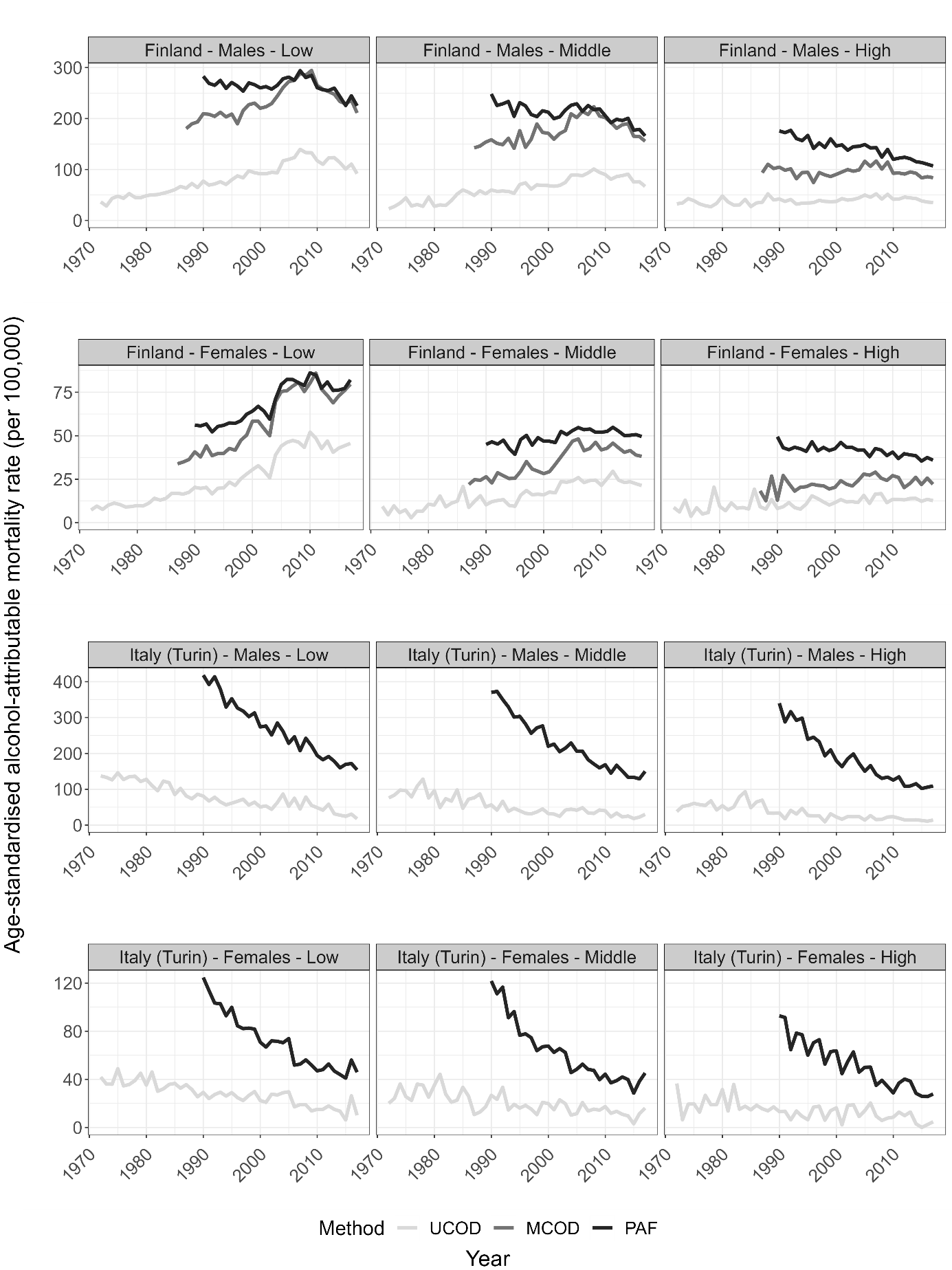


**Fig S2.3a. Age patterns in alcohol attributable mortality according to existing estimation methods by sex, for the general population and by educational level for those aged 30 and older, Finland 1990-2017 in five 5-year intervals (excl. 2015-2017).** UCOD = ‘Underlying cause of death’, MCOD = ‘Multiple cause of death’, PAF = ‘Population-attributable fractions-based’; Rates are expressed per 100,000 person years. The Y-axis scale differs by sex to improve visibility of the results. Data source: Statistics Finland.


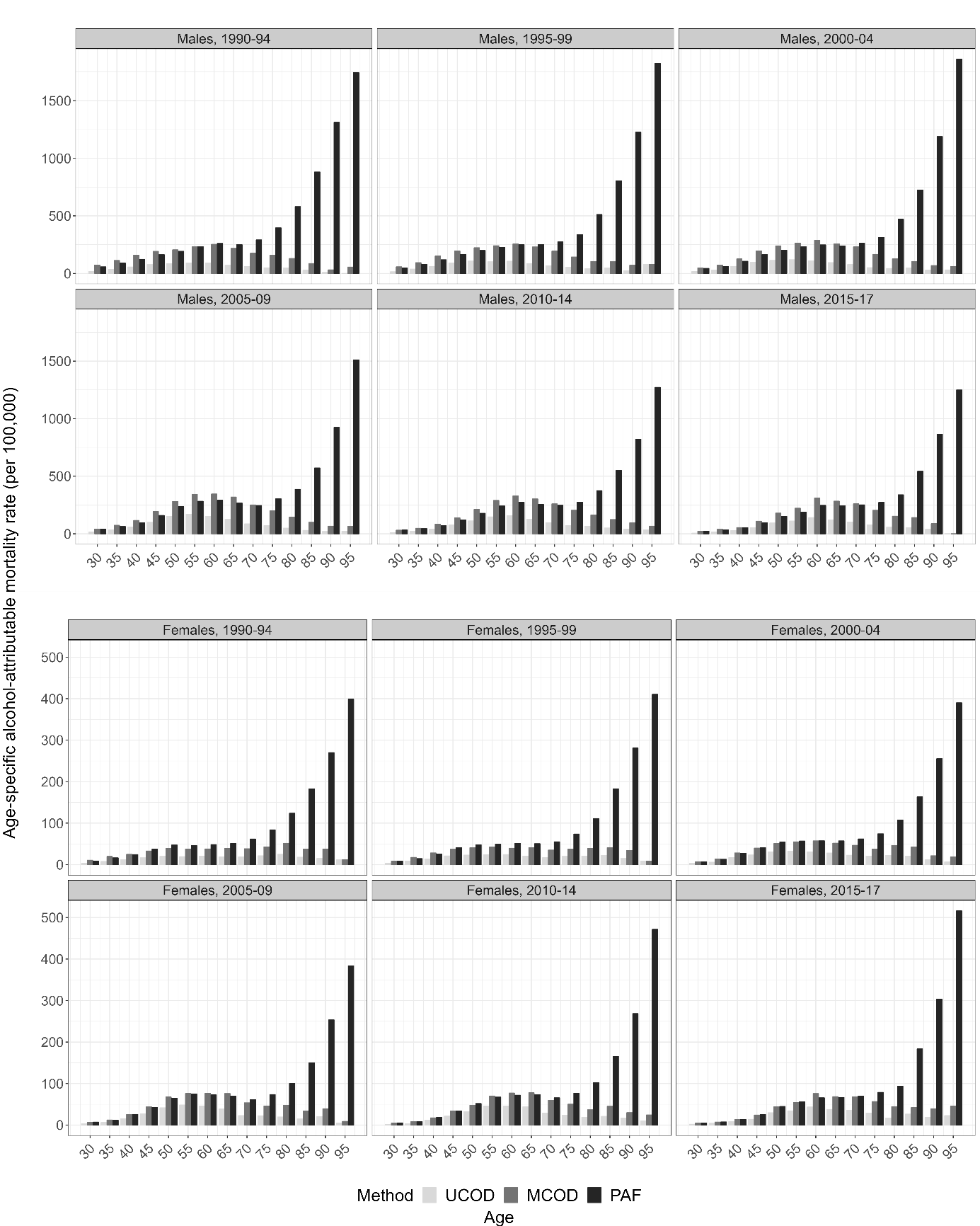


**Fig S2.3b. Age patterns in alcohol attributable mortality according to existing estimation methods by sex, for the general population and by educational level, Italy (Turin) 1990-2017 in five 5-year intervals (excl. 2015-2017).** UCOD = ‘Underlying cause of death’, PAF = ‘Population-attributable fractions-based’; Rates are expressed per 100,000 person years. The Y-axis scale differs by sex to improve visibility of the results. Data source: Turin Longitudinal Study.


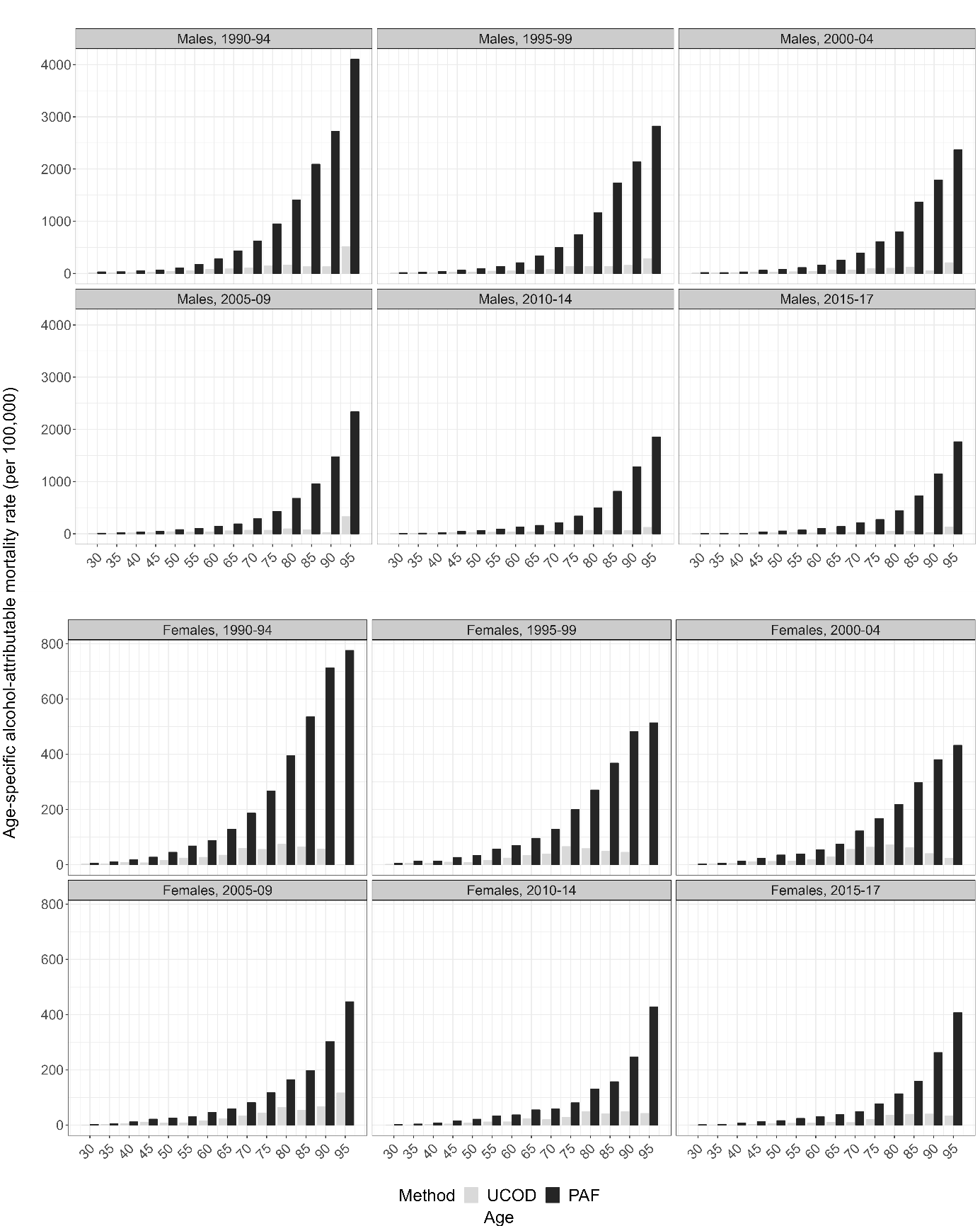


**Fig S2.4.** **Trends in new age-standardised mortality rates in alcohol-attributable mortality compared to other available estimation methods for alcohol attributable mortality by country and sex, for the general population and by educational level, ages 30 and older, Finland & Italy (Turin), 1972-2017.** UCOD = ‘Underlying cause of death’, MCOD = ‘Multiple cause of death’, PAF = ‘Population-attributable fractions-based’, UCOD-Enh. = ‘Enhanced underlying cause of death’; Rates are expressed per 100,000 person years. The Y-axis scale differs by sex and country to improve visibility of the results. Data sources: Statistics Finland, Turin Longitudinal Study.


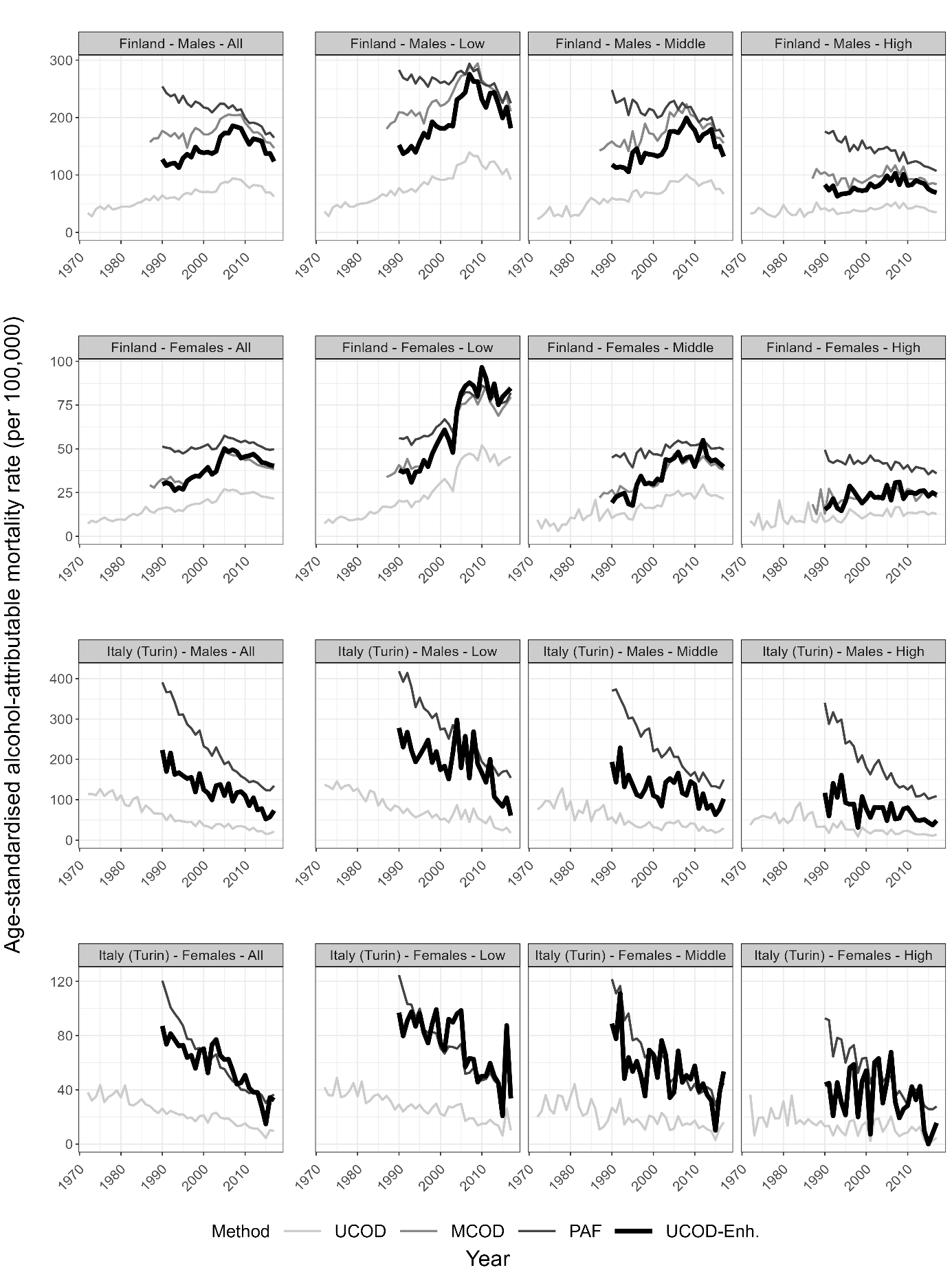


**Fig S2.5a. Age patterns in alcohol attributable mortality according to all estimation methods by sex for the general population aged 30 and older, Finland 1990-2017 in five 5-year intervals (excl. 2015-2017).** UCOD = ‘Underlying cause of death’, MCOD = ‘Multiple cause of death’, UCOD-Enh. = ‘Enhanced underlying cause of death’; Rates are expressed per 100,000 person years. The Y-axis scale differs by sex to improve visibility of the results. Data source: Statistics Finland.


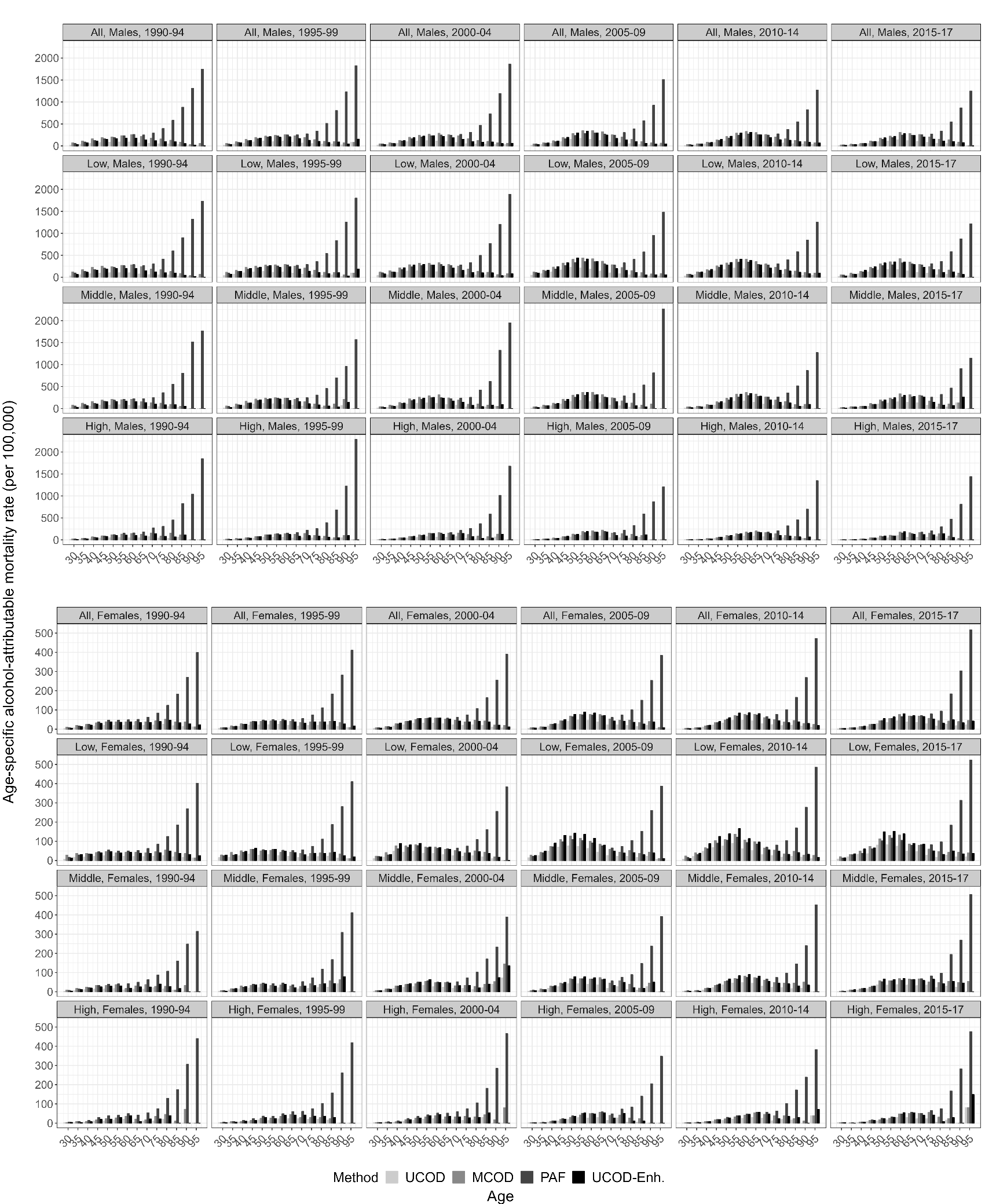


**Fig S2.5b. Age patterns in alcohol attributable mortality according to all estimation methods by sex for the general population aged 30 and older, Italy (Turin) 1990-2017 in five 5-year intervals (excl. 2015-2017).** UCOD = ‘Underlying cause of death’, PAF = ‘Population-attributable fractions-based’, UCOD-Enh. = ‘Enhanced underlying cause of death’; Rates are expressed per 100,000 person years. The Y-axis scale differs by sex to improve visibility of the results. Data source: Turin Longitudinal Study.


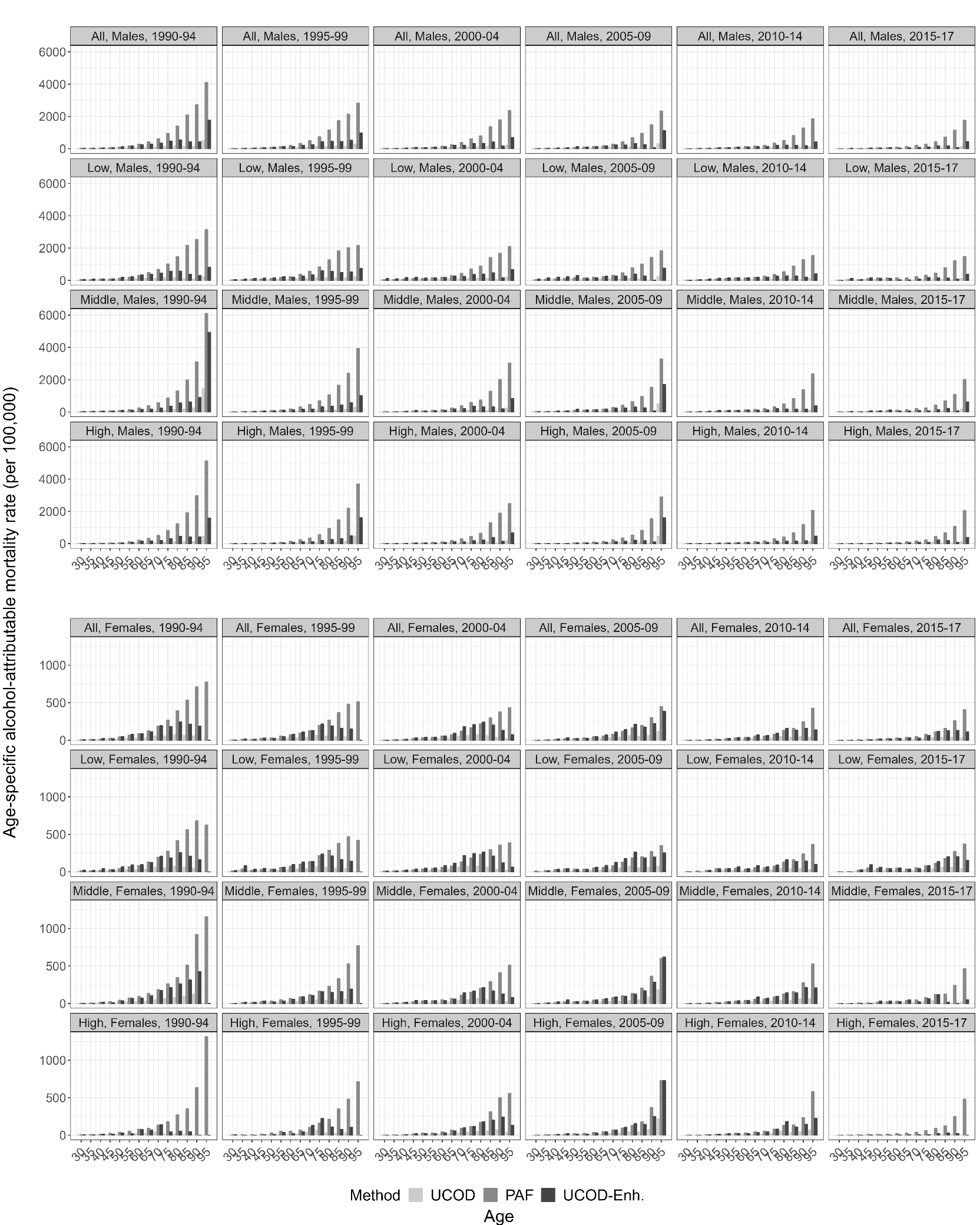


**Fig S2.6. Trends in relative educational inequalities (relative inequality index) in alcohol-attributable mortality by country and sex for ages 30 and older according to different estimation methods, including a newly developed one, 1972-2017.** Dots represent observed values, lines are smoothed trends using cubic splines. UCOD = ‘Underlying cause of death’, MCOD = ‘Multiple cause of death’, PAF = ‘Population attributable fractions-based’, UCOD-Enh. = ‘Enhanced underlying cause of death’; SII expressed per 100,000 person years; Outliers (RII>8) among Italian females for the UCOD and UCOD-Enh. methods in 1987, 1998, 2004, 2015, and 2016 were excluded to enhance visibility of the overall figure; Data sources: Statistics Finland, Turin Longitudinal Study.

**
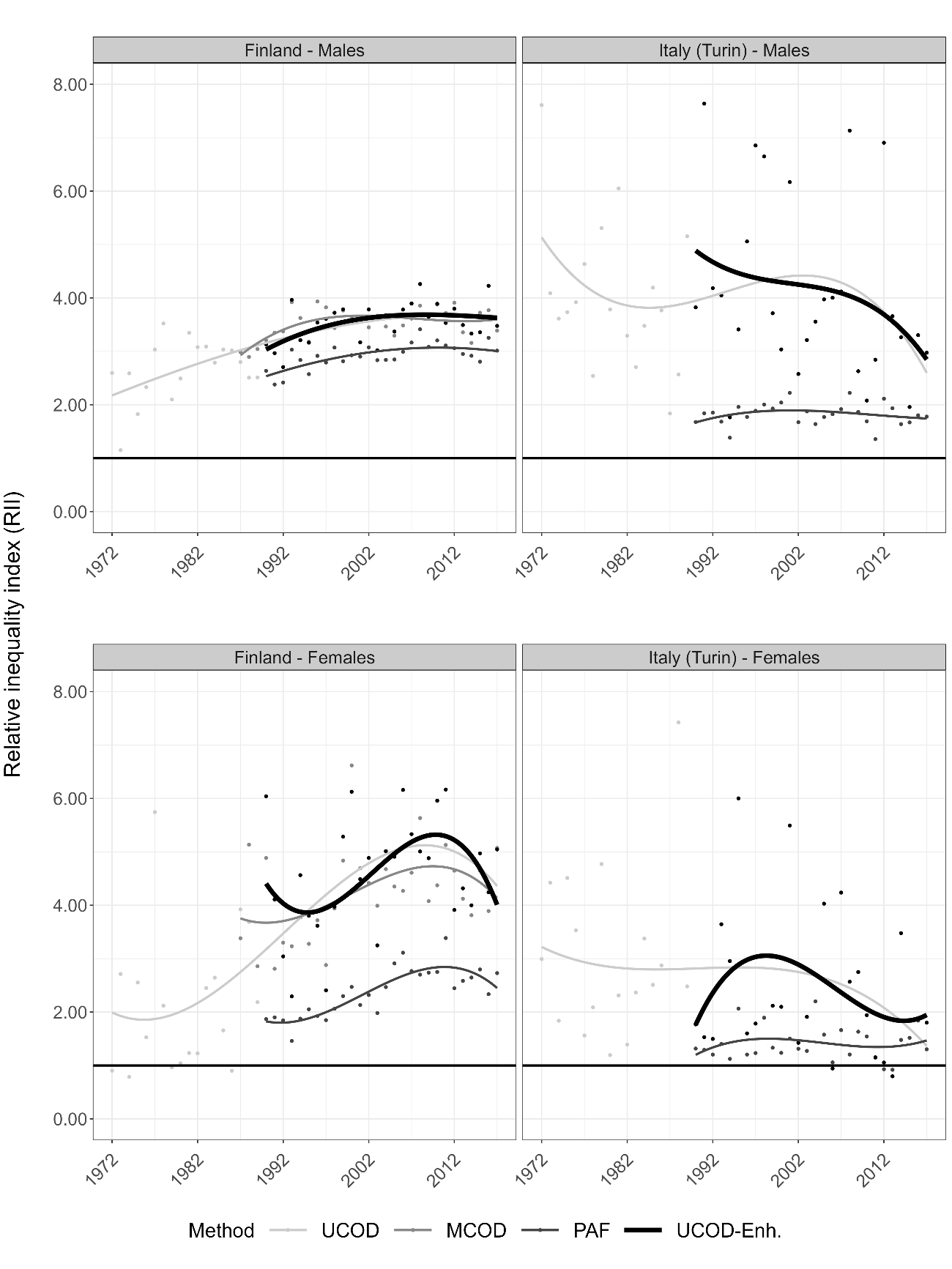
**

**Fig S2.7. Trends in absolute educational inequalities (slope index of inequality) in alcohol-attributable mortality by country and sex for ages 30 and older according to different estimation methods, including a newly developed one, 1972-2017.** Dots represent observed values, lines are smoothed trends using cubic splines. UCOD = ‘Underlying cause of death’, MCOD = ‘Multiple cause of death’, PAF = ‘Population attributable fractions-based’, UCOD-Enh. = ‘Enhanced underlying cause of death’; SII expressed per 100,000 person years; Data sources: Statistics Finland, Turin Longitudinal Study.

**
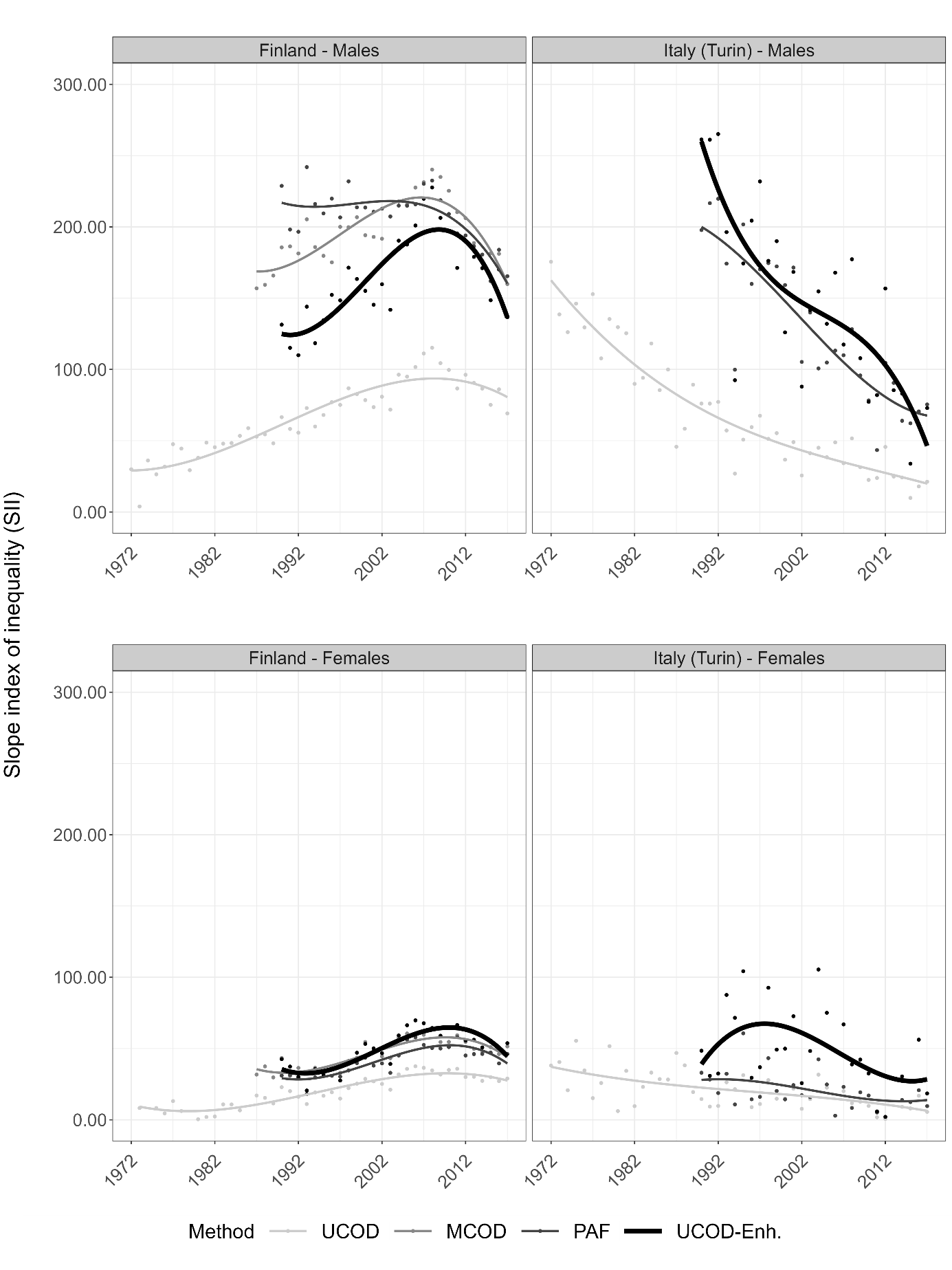
**

**REFERENCES**

[1] van de Luitgaarden IAT, van Oort S, Bouman EJ, Schoonmade LJ, Schrieks IC, Grobbee DE, et al. Alcohol consumption in relation to cardiovascular diseases and mortality: a systematic review of Mendelian randomization studies. European Journal of Epidemiology. 2021;37(7):655-69.

[2] IHME. Global Burden of Disease Collaborative Network. Global Burden of Disease Study 2017 (GBD 2017). Results. Seattle, United States Institute for Health Metrics and Evaluation (IHME); 2018 [Available from: <http://ghdx.healthdata.org/gbd-results-tool>.

[3] Mackenbach JP, Kulhanova I, Bopp M, Borrell C, Deboosere P, Kovacs K, et al. Inequalities in Alcohol-Related Mortality in 17 European Countries: A Retrospective Analysis of Mortality Registers. PLoS Medicine. 2015;12(12):e1001909.

[4] Trias-Llimós S, Martikainen P, Mäkelä P, Janssen F. Comparison of different approaches for estimating age-specific alcohol-attributable mortality: The cases of France and Finland. PLoS ONE. 2018;13(3):1.

[5] Durkin A, Connolly S, O’Reilly D. Quantifying Alcohol-Related Mortality: Should Alcohol-Related Contributory Causes of Death be Included? Alcohol and Alcoholism. 2010;45(4):374-8.

[6] Manthey J, Rehm J. Mortality from Alcoholic Cardiomyopathy: Exploring the Gap between Estimated and Civil Registry Data. Journal of Clinical Medicine. 2019;8(8):1137.

[7] Janssen F, El Gewily S, Bardoutsos A, Trias-Llimós S. Past and Future Alcohol-Attributable Mortality in Europe. International Journal of Environmental Research and Public Health. 2020;17(23).

[8] European Commission. Revision of the European Standard Population - Report of Eurostat's task force. Luxembourg: Publications Office of the European Union; 2013.

1. A version that allows for a beneficial effect of mild alcohol consumption on cardiovascular disease exists in theory (I.e. negative AFs for a number of causes in specific age groups). However, this so-called “cardioprotective effect” is highly contested [1]. Although negative AFs for diabetes are also common within the PAF method, we replace any negative death counts in country-sex-education-age-strata by 0. [↑](#footnote-ref-1)
