# Supplementary material for: The impact of estimation methods for alcohol-attributable mortality on long-term trends for the general population and by educational level in Finland and Italy (Turin)
Source: PLoS One. 2023 Dec 14;18(12):e0295760. doi: 10.1371/journal.pone.0295760 (PMC10721192; doi:10.1371/journal.pone.0295760)

Supporting information 3: additional figures

**Fig S3.1a. Age patterns in alcohol-attributable mortality by country, sex, and educational level according to different estimation methods for ages 30 and older, clubbed rates by five year period (excl. 2016-17), 1990 - 2017, Finland.** UCOD = ‘Underlying cause of death’, MCOD = ‘Multiple cause of death’, PAF = ‘Population attributable fractions-based’, UCOD-Enh. = ‘Enhanced underlying cause of death’; Rates expressed per 100,000 person years; The Y-axis scale differs by sex to improve visibility of the results; Data source: Statistics Finland.


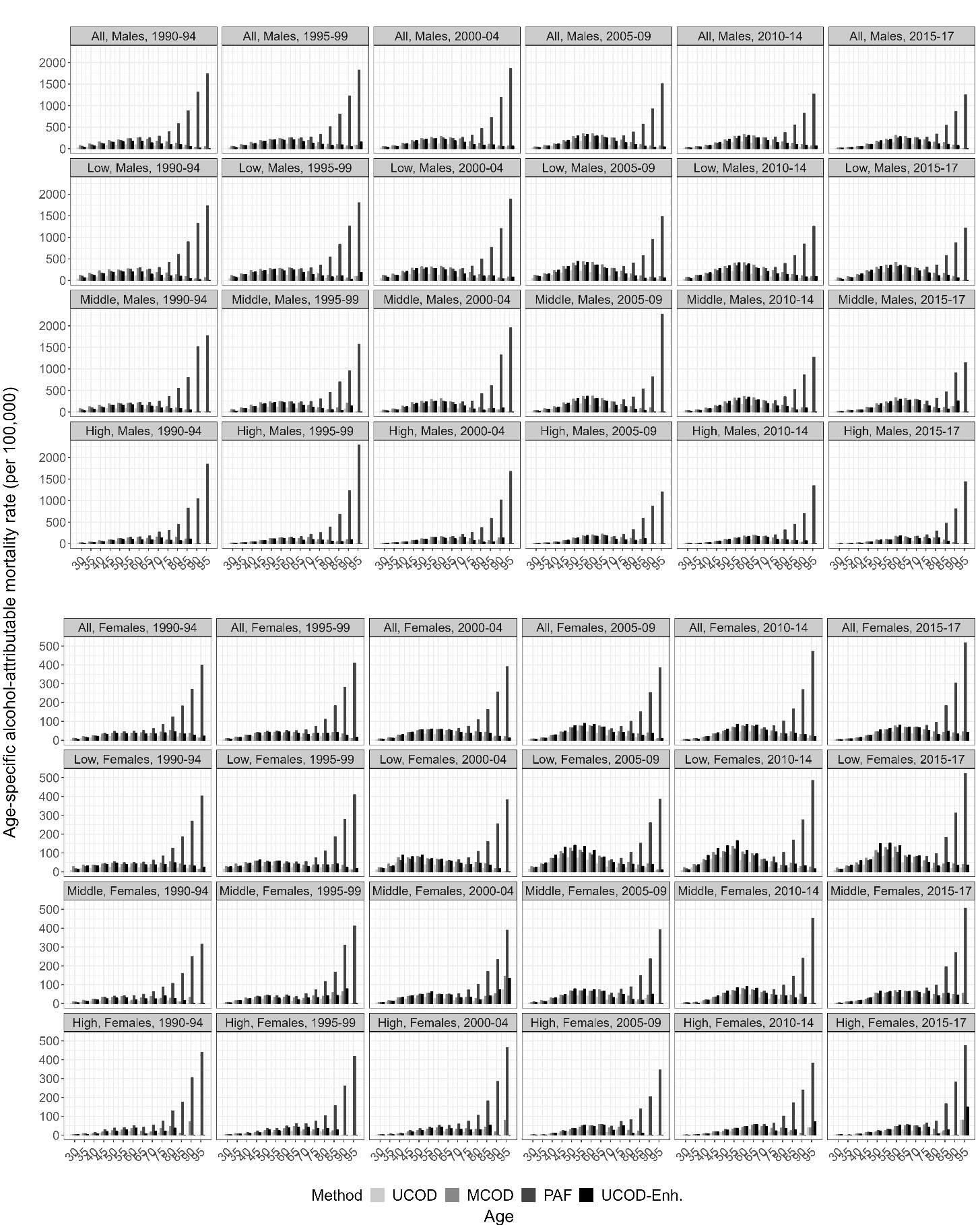


**Fig S3.1b. Age patterns in alcohol-attributable mortality by country, sex, and educational level according to different estimation methods for ages 30 and older, clubbed rates by five year period (excl. 2016-17), 1990 - 2017, Italy (Turin).** UCOD = ‘Underlying cause of death’, PAF = ‘Population attributable fractions-based’, UCOD-Enh. = ‘Enhanced underlying cause of death’; Rates expressed per 100,000 person years; The Y-axis scale differs by sex to improve visibility of the results; Data source: Turin Longitudinal Study.


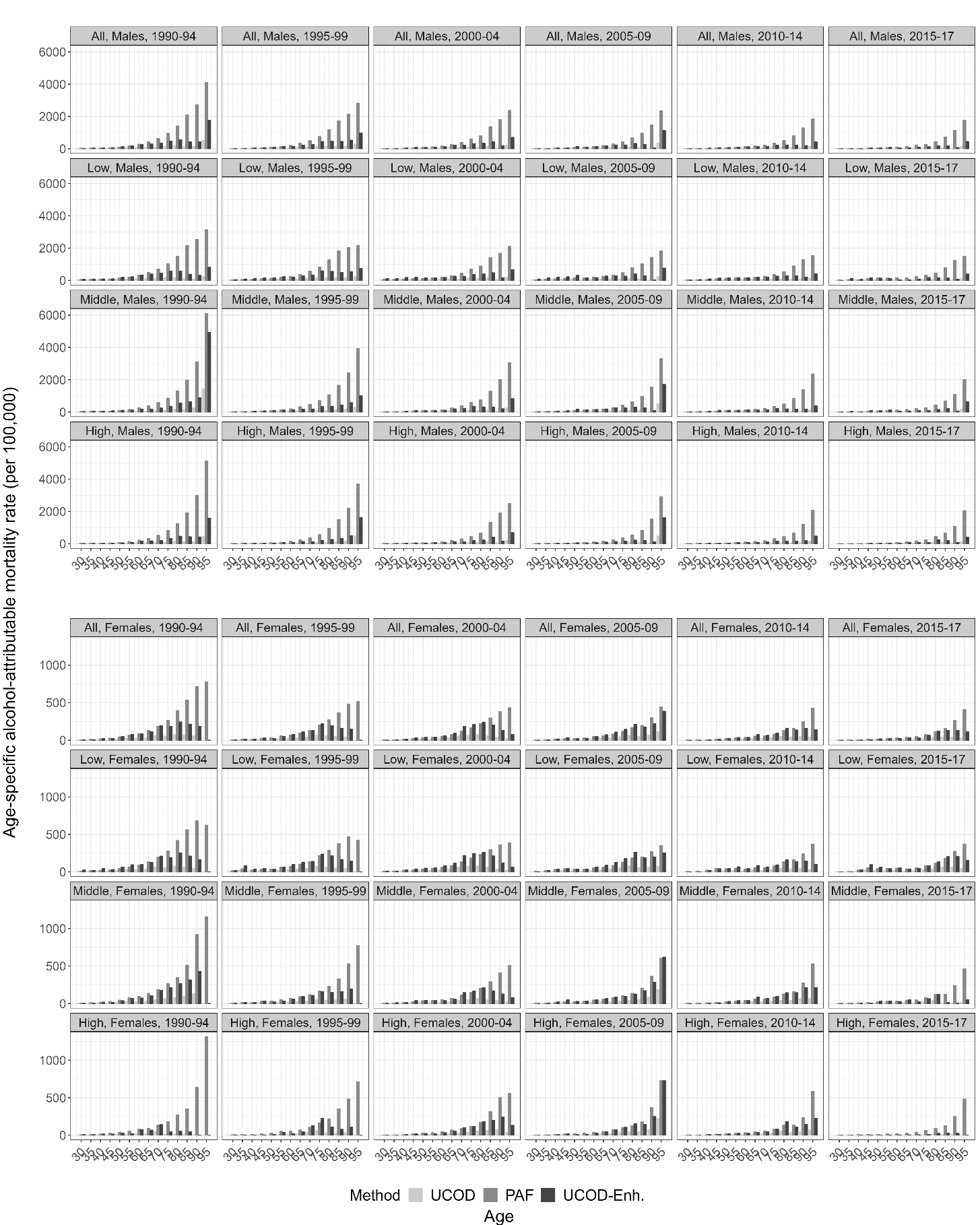


**Fig S3.2. Trends in age-standardised alcohol-attributable mortality rates by country, sex, and educational level according to different estimation methods, including a newly developed one, 1972-2017.** UCOD = ‘Underlying cause of death’, MCOD = ‘Multiple cause of death’, PAF = ‘Population attributable fractions-based’, UCOD-Enh. = ‘Enhanced underlying cause of death’; Rates expressed per 100,000 person years; The Y-axis scale differs by sex to improve visibility of the results; Data sources: Statistics Finland, Turin Longitudinal Study.


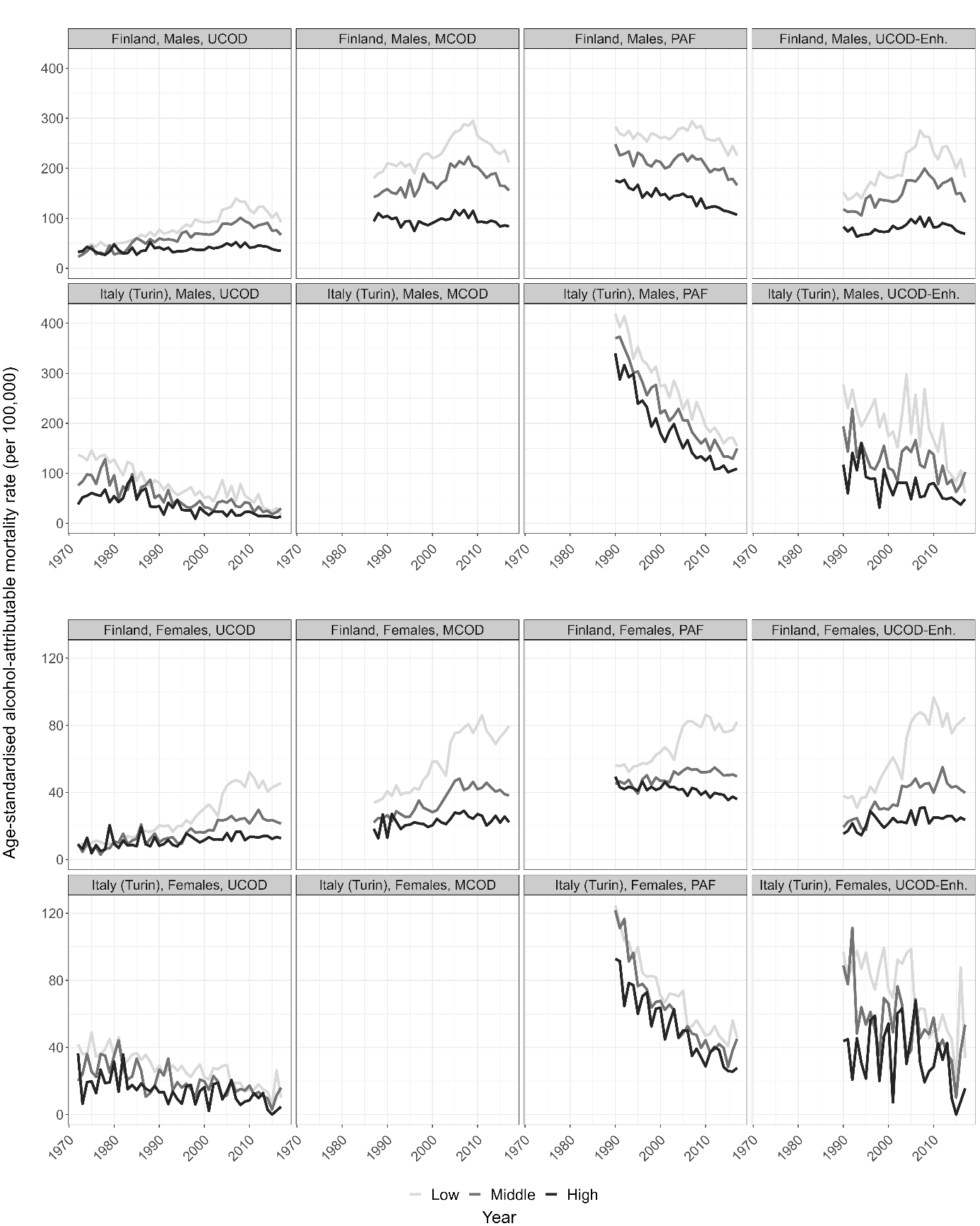


**Fig S3.3a. Trends in age-standardised alcohol-attributable mortality rates for cause of death groups within the underlying cause of death (UCOD) method by country, sex, and educational level for those aged 30 and older, 1972-2017.** CVD = ‘Cardiovascular disease’; Rate expressed per 100,000 person years; The Y-axis scale differs by sex to improve visibility of the results; Data sources: Statistics Finland, Turin Longitudinal Study.


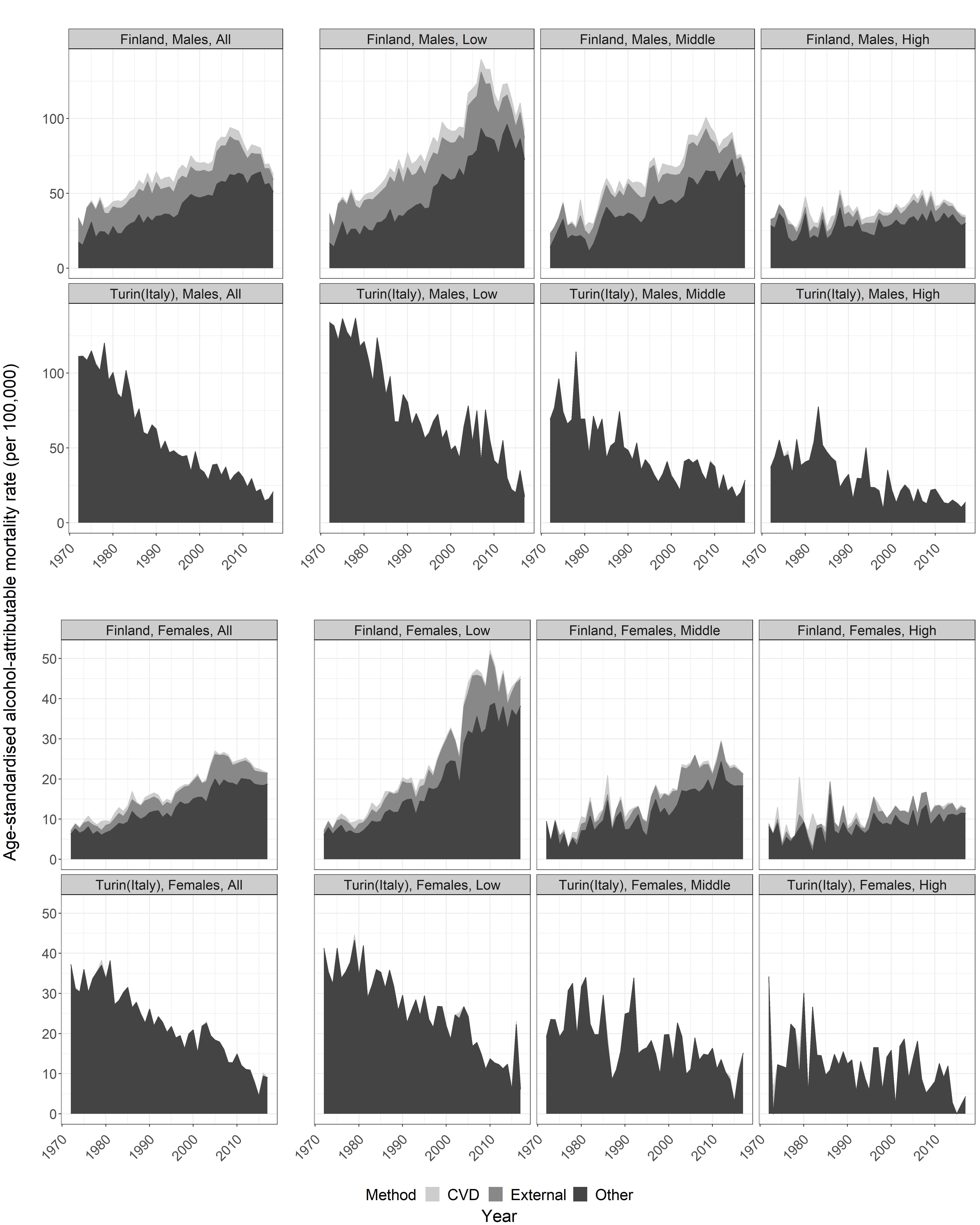


**Fig S3.3b. Trends in age-standardised alcohol-attributable mortality rates for cause of death groups within the Multiple cause of death (MCOD) method in Finland by sex and educational level for those aged 30 and older, 1987-2017.** CVD = ‘Cardiovascular disease’; Rate expressed per 100,000 person years; The Y-axis scale differs by sex to improve visibility of the results; Data sources: Statistics Finland.

**
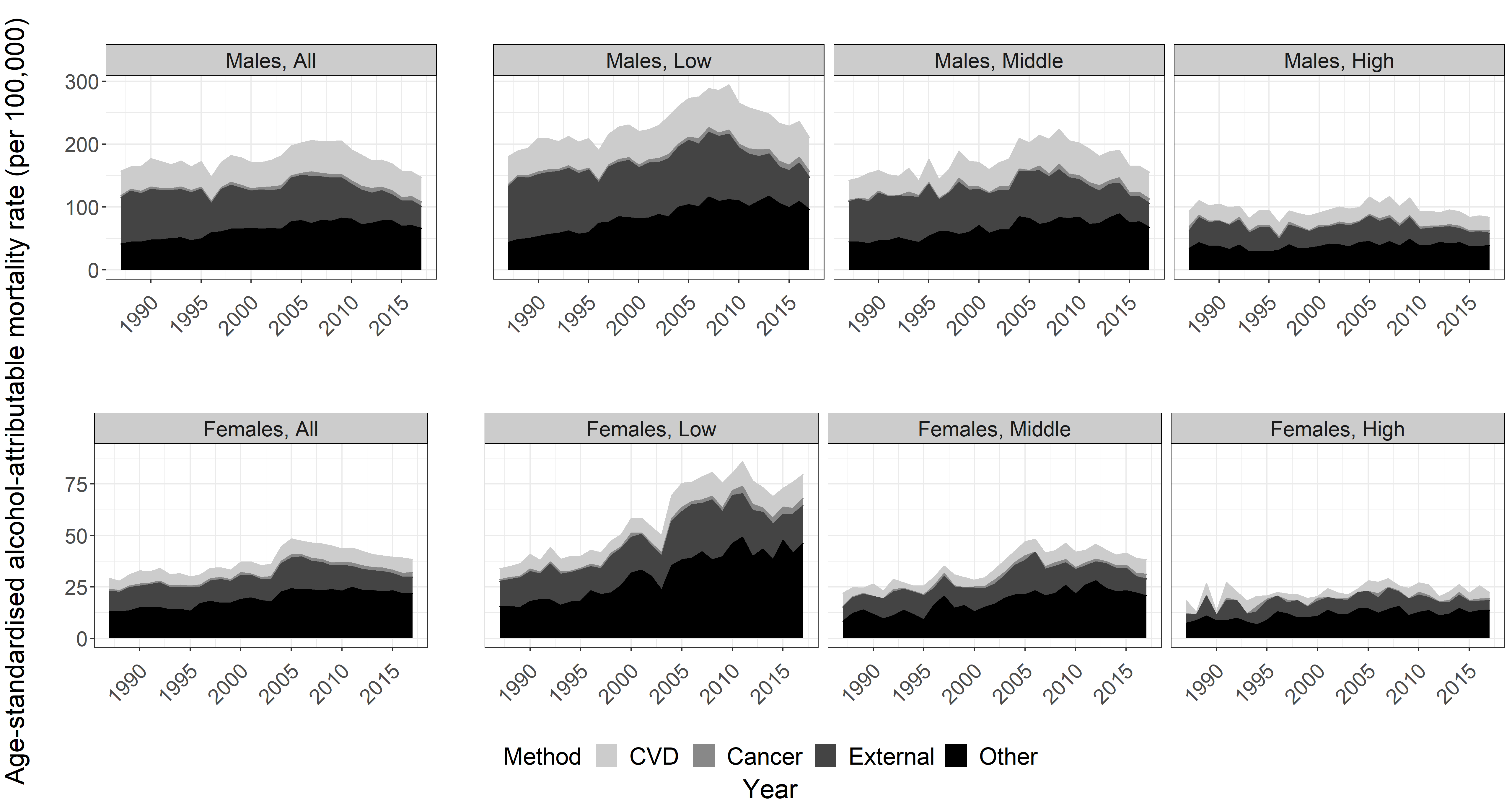
**

**Fig S3.3c. Trends in age-standardised alcohol-attributable mortality rates for cause of death groups within the Population attributable fractions- based (PAF) method by country, sex, and educational level for those aged 30 and older, 1972-2017.** CVD = ‘Cardiovascular disease’; Rate expressed per 100,000 person years; The Y-axis scale differs by sex to improve visibility of the results; Data sources: Statistics Finland, Turin Longitudinal Study.


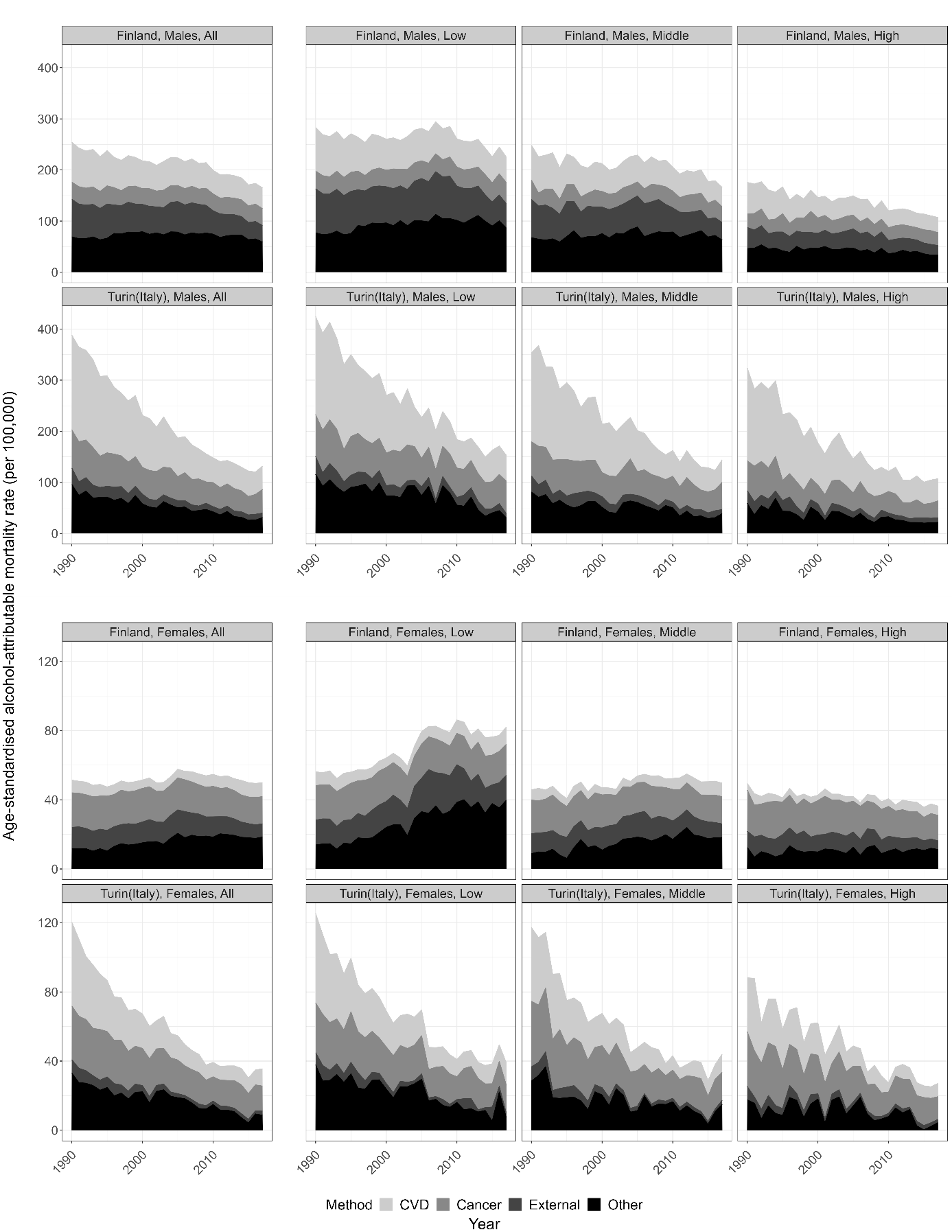

Supplement: S3 File — (DOCX) [file pone.0295760.s003.docx]
